# Supplementary material for: How Do Users Respond to Mass Vaccination Centers? A Cross-Sectional Study Using Natural Language Processing on Online Reviews to Explore User Experience and Satisfaction with COVID-19 Vaccination Centers
Source: Vaccines (Basel). 2023 Jan 9;11(1):144. doi: 10.3390/vaccines11010144 (PMC9861127; doi:10.3390/vaccines11010144)
Supplement: Supplementary file 1 [file vaccines-11-00144-s001.zip › Appendix A_Vaccines_User Experience Vaccination Center Online Reviews.docx]

**Appendix A**


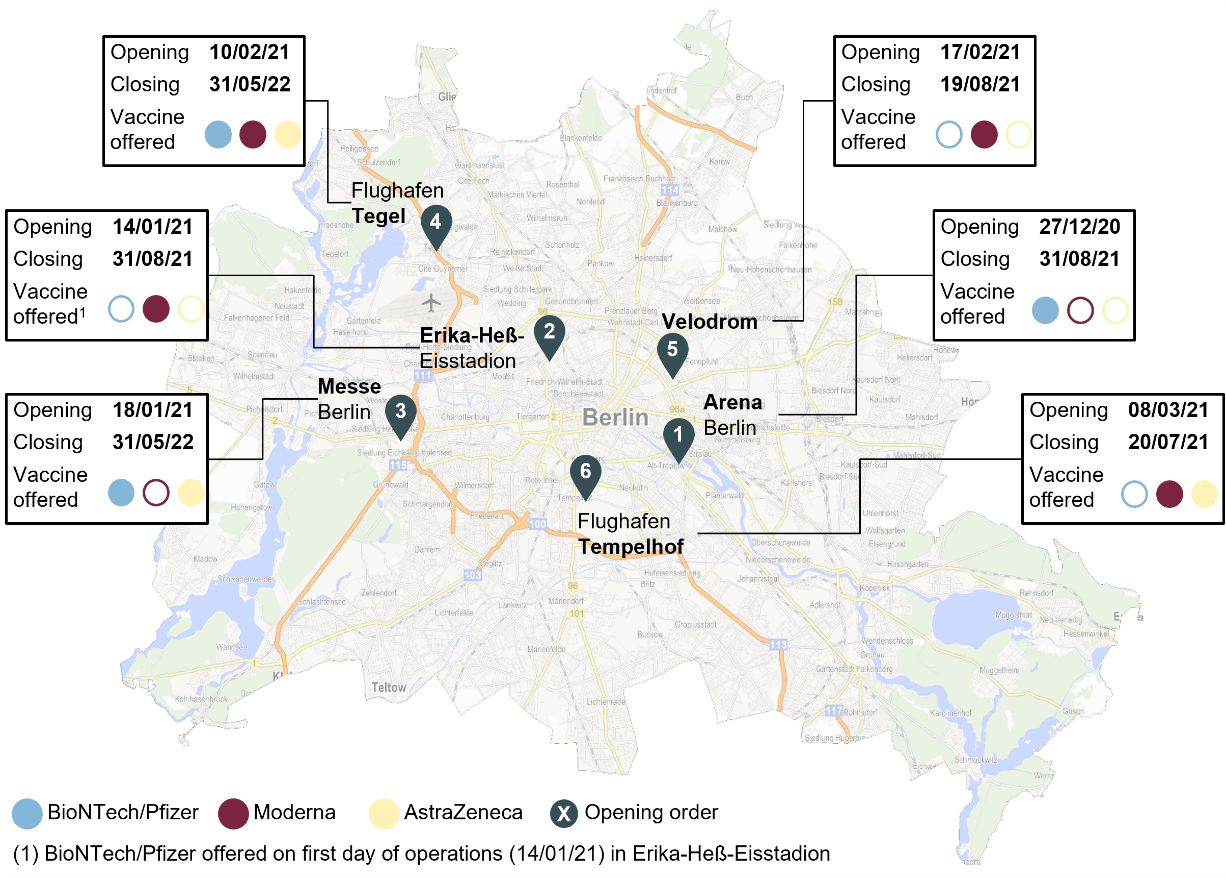


**Figure A1:** Overview of COVID-19 vaccination centers in Berlin.

A total of six mass vaccination centers were set up in Berlin and opened between December 27, 2020, and March 8, 2021. The centers were set up by or on behalf of the federal states and financed through public funds[1]. In Berlin, the centers were operated by several Berlin-based humanitarian aid organizations coordinated through DRK SWB, a non-profit company established by the German Red Cross Berlin, the Association of Statutory Health Insurance Physicians Berlin and the Senate Department for Health, Nursing and Equality. The vaccination centers were spread across the city to ensure reachability from all districts. The vaccination centers were open daily usually between 9AM and 5PM with a few exceptional closures (e.g., two sites closed temporarily due to a suspension of AstraZeneca while side effects were being reviewed[2,3]). A single vaccination center could perform up to 4.000 vaccinations per day[4]. The effective daily volume depended on the number of vaccines available, and the number of appointments booked. Three different vaccines were available at the vaccination centers. In the beginning vaccinations were offered based on appointments and priority group, from July 2021 walk-ins were introduced[5]. As the vaccination campaign progressed and other structures (e.g., outpatient practices and company physicians) got involved, vaccination centers were gradually closed. At the time the study was conducted two vaccination centers were still running.

1 Bundesministerium für Gesundheit. Verordnung zum Anspruch auf Schutzimpfung gegen das Coronavirus SARS-CoV-2 (Coronavirus-Impfverordnung – CoronaImpfV). 2021.

2 Impfbetrieb in Tegel und Tempelhof startet am Freitag wieder. Berlin.de - Das offizielle Hauptstadtportal 2021. https://www.berlin.de/aktuelles/berlin/6479694-958092-impfbetrieb-in-tegel-und-tempelhof-start.html

3 Senatsverwaltung für Gesundheit, Pflege und Gleichstellung (2016-2021). Berlin setzt Impfen mit AstraZeneca aus. Berlin: 2021. https://www.berlin.de/sen/archiv/gpg-2016-2021/2021/pressemitteilung.1064782.php (accessed 12 Apr 2022).

4 Bauer C. Corona in Berlin: Impfzentrum auf dem Messegelände bleibt bis Februar auf. Berl. Morgenpost. 2021.https://www.morgenpost.de/bezirke/charlottenburg-wilmersdorf/article233947755/Impfzentrum-an-der-Messe-bleibt.html (accessed 12 Apr 2022).

5 Senatsverwaltung für Gesundheit, Pflege und Gleichstellung (2016-2021). Impfen ohne Termin jetzt in allen Berliner Impfzentren möglich – zusätzlich zu Moderna auch Biontech. Berlin: : Senatsverwaltung für Gesundheit, Pflege und Gleichstellung (2016-2021) 2021. https://www.berlin.de/sen/archiv/gpg-2016-2021/2021/pressemitteilung.1111392.php
