# Supplementary material for: How Do Users Respond to Mass Vaccination Centers? A Cross-Sectional Study Using Natural Language Processing on Online Reviews to Explore User Experience and Satisfaction with COVID-19 Vaccination Centers
Source: Vaccines (Basel). 2023 Jan 9;11(1):144. doi: 10.3390/vaccines11010144 (PMC9861127; doi:10.3390/vaccines11010144)
Supplement: Supplementary file 1 [file vaccines-11-00144-s001.zip › Appendix B_Vaccines_User Experience Vaccination Center Online Reviews_Revision 1.docx]

**Appendix B**

The raw data was obtained on 27 dec 2021 from Google Maps Reviews public review websites (Table B1) using Outscraper (<https://outscraper.com>). Online reviews for all six vaccination centers in Berlin were obtained for the period 27 dec 2020 00:00:00 to 27 dec 2021 00:00:00. The dataset used for the analysis can be found on github at: <https://github.com/stellaroxanne/User-Reviews-Vaccination-Centers-Berlin>.

**Table B1:** Links to Google Maps Online Review sites for the vaccination centers analyzed

| **Name Vaccination Center** | **Google Maps Online Review Link** |
| --- | --- |
| Arena | <https://www.google.com/maps/place/Arena+Berlin/@52.4964613,13.4543597,15z/data=!4m5!3m4!1s0x0:0x6852fd9350063186!8m2!3d52.4964613!4d13.4543597> |
| Erika-Heß-Eisstadion | <https://www.google.com/maps/place/Impfzentrum+Berlin+-+Erika-He%C3%9F-Eisstadion/@52.537194,13.3674287,17z/data=!3m2!4b1!5s0x47a8518fec26cc1b:0x9d079a3d045872a3!4m5!3m4!1s0x47a8516968c04057:0x759b414ae8c704fc!8m2!3d52.537194!4d13.3696174> |
| Flughafen Tegel | <https://www.google.com/maps/place/Impfzentrum+Berlin+Tegel+Terminal+C/@52.555381,13.293457,17z/data=!3m1!4b1!4m5!3m4!1s0x47a857e0f5b20727:0x27042a6252ee947c!8m2!3d52.555381!4d13.2956457> |
| Flughafen Tempelhof | <https://www.google.com/maps/place/Impfzentrum+Flughafen+Tempelhof/@52.4827589,13.3909385,17z/data=!3m1!4b1!4m5!3m4!1s0x47a84f62dae9d565:0xd5b04abca92add1f!8m2!3d52.4827589!4d13.3931272> |
| Messe | <https://www.google.com/maps/place/Corona-Impfzentrum+Messe+Berlin/@52.5063417,13.2704876,17z/data=!3m1!4b1!4m5!3m4!1s0x47a8571f61b570a7:0x6db977b39837a08a!8m2!3d52.5063417!4d13.2726763> |
| Velodrom | <https://www.google.com/maps/place/Impfzentrum+Berlin+-+Velodrom/@52.5305156,13.4486547,17z/data=!3m1!4b1!4m5!3m4!1s0x47a84fc3a22029cf:0x2e9371eb78584ce8!8m2!3d52.5305156!4d13.4508434> |
