# Supplementary material for: How Do Users Respond to Mass Vaccination Centers? A Cross-Sectional Study Using Natural Language Processing on Online Reviews to Explore User Experience and Satisfaction with COVID-19 Vaccination Centers
Source: Vaccines (Basel). 2023 Jan 9;11(1):144. doi: 10.3390/vaccines11010144 (PMC9861127; doi:10.3390/vaccines11010144)
Supplement: Supplementary file 1 [file vaccines-11-00144-s001.zip › Appendix C_Vaccines_User Experience Vaccination Center Online Reviews.docx]

**Appendix C**

The figure below (Figure C1) shows the final framework for vaccination center user experience composed of user journey and key determinants.

The user journey follows the path of a vaccination center user from their invitation to get vaccinated up to their follow-up appointment. It is composed of three phases: pre-visit, visit and post-visit. The user journey can also be applied to vaccinations in other settings as it follows a similar overarching path.

The table below (Table C1) shows the different publications on vaccination centers and patient reported outcome and experience measures that were referenced to determine the relevant key determinants for a positive vaccination experience. The key determinants that form part of the final framework can be clustered into three groups: staff, process/management and location.


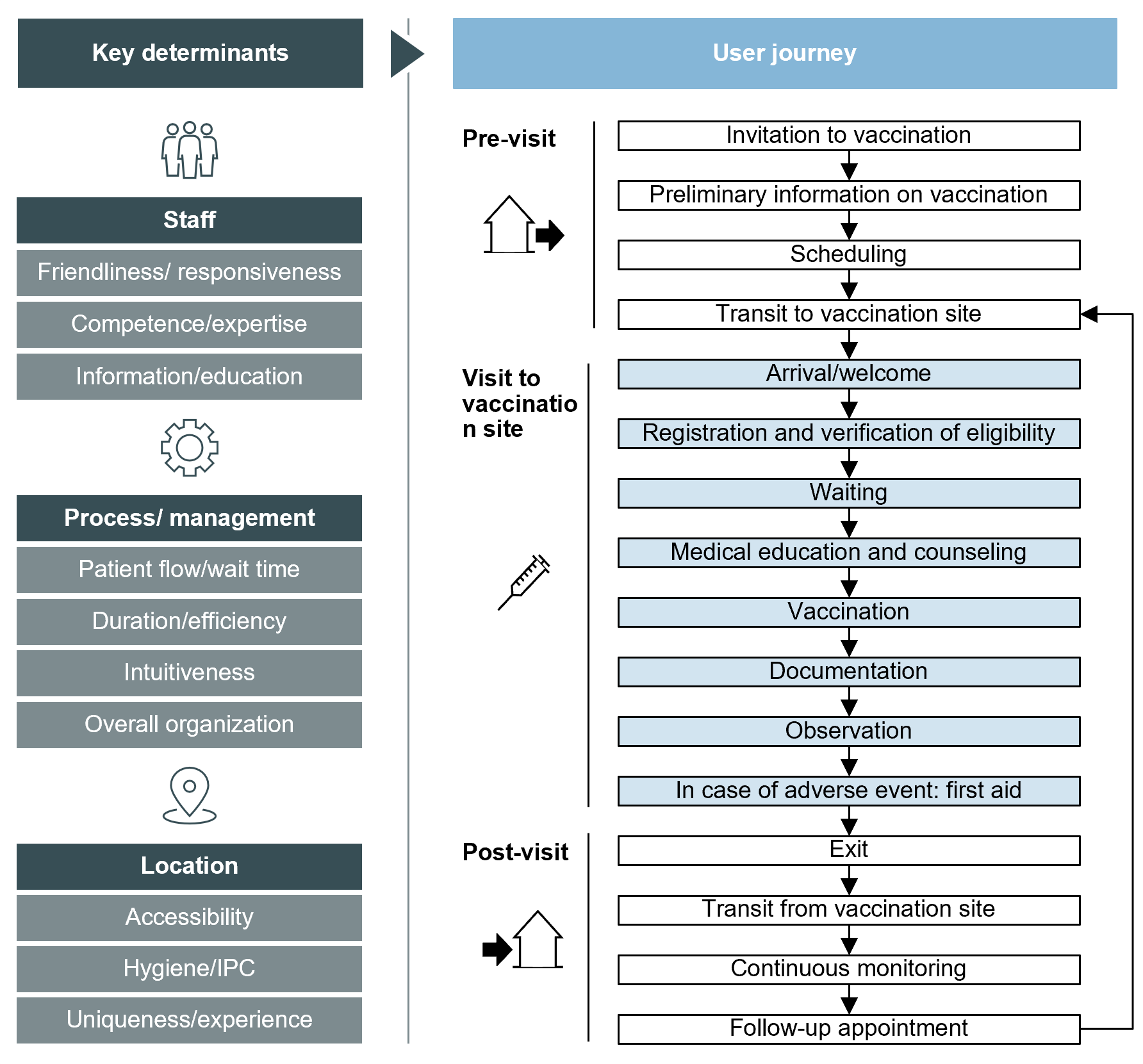


**Figure C1:** Framework for vaccination center user experience including key determinants of user experience and the vaccination center user journey.

**Table 2.** Key determinants of user experience as referred to in publications on vaccination centers or reference publications on patient reported experience measures (PREMs) and patient reported outcome measures (PROMs).

| **Fields** | **Key determinants** | **Final framework** | **MoH Handbook (2020) [37]** | **Golberg et al. (2021) [38]** | **Goralnick et al. (2021)[39]** | **OECD (2018)[40]** | **Bertelsmann (2010)[41]** |
| --- | --- | --- | --- | --- | --- | --- | --- |
| **Staff** | Friendliness /  responsiveness | **x** |  | x |  | x | x |
|  | Competence /  expertise | **x** | x |  |  |  | x |
|  | Information /  education | **x** | x |  |  | x | x |
| **Process /**  **Management** | Patient flow /  wait time | **x** | x | x |  | x | x |
|  | Duration /  efficiency | **x** |  |  |  | x |  |
|  | Intuitiveness | **x** | x | x |  | x | x |
|  | Overall organization | **x** | x | x |  |  |  |
| **Location** | Accessibility | **x** | x | x | x |  | x |
|  | Hygiene/ IPC | **x** | x | x |  |  | x |
|  | Uniqueness | **x** |  | x |  |  |  |
|  | Reputation | **-** |  |  |  |  |  |
| **Patient choice** | Vaccine availability | **-** |  |  |  |  |  |
|  | Cost | **-** |  |  | x | x |  |
