# Supplementary material for: How Do Users Respond to Mass Vaccination Centers? A Cross-Sectional Study Using Natural Language Processing on Online Reviews to Explore User Experience and Satisfaction with COVID-19 Vaccination Centers
Source: Vaccines (Basel). 2023 Jan 9;11(1):144. doi: 10.3390/vaccines11010144 (PMC9861127; doi:10.3390/vaccines11010144)
Supplement: Supplementary file 1 [file vaccines-11-00144-s001.zip › manuscript_2112191_v7_SD.docx]

Article

How Do Users Respond to Mass Vaccination Centers?
A Cross-Sectional Study Using Online Reviews to Explore User Experience and Satisfaction with COVID-19 Vaccination
Centers

| **Citation:** Danek, S.; Büttner, M.; Krois, J.; Schwendicke, F. How Do Users Respond to Mass Vaccination Centers? A Cross-Sectional Study Using Online Reviews to Explore User Experience and Satisfaction with COVID-19 Vaccination Centers. *Vaccines* **2023**, *11*, x. https://doi.org/10.3390/xxxxx  Academic Editor(s):  Received: 5 December 2022  Revised: 21 December 2022  Accepted: date  Published: date  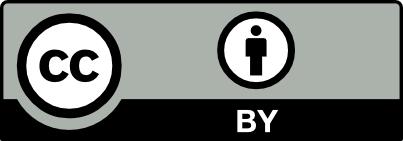  **Copyright:** © 2022 by the authors. Submitted for possible open access publication under the terms and conditions of the Creative Commons Attribution (CC BY) license (https://creativecommons.org/licenses/by/4.0/). |
| --- |

Stella Danek *, Martha Büttner, Joachim Krois and Falk Schwendicke

Charité—Universitätsmedizin Berlin, 10117 Berlin, Germany; martha.buettner@charite.de (M.B.); joachim.krois@gmail.com (J.K.); falk.schwendicke@charite.de (F.S.)

***** Correspondence: [stella.danek@charite.de](mailto:stella.danek@charite.de) (S.D.)

**Abstract:** To reach large groups of vaccine recipients, several high-income countries introduced mass vaccination centers for COVID-19. Understanding user experiences of these novel structures can help optimize their design and increase patient satisfaction and vaccine uptake. This study employed user online reviews of vaccination centers to gauge user experience and identify its key determinants over time, by sentiment, and by interaction. Machine learning methods were used to analyze a comprehensive set of Google reviews of six COVID-19 mass vaccination centers in Berlin from December 2020 to December 2021. We included 3647 user online reviews in the analysis. Of these, 89% (3261/3647) were positive according to user rating (four to five of five stars). A total of 85% (2740/3647) of all reviews contained text. Topic modeling of the reviews containing text identified five optimally latent topics, and keyword extraction identified 47 salient keywords. The most important themes were organization, friendliness/responsiveness, and patient flow/wait time. Key interactions for users of vaccination centers included waiting, scheduling, transit, and the vaccination itself. Keywords connected to scheduling and efficiency, such as “appointment” and “wait”, were most prominent in negative reviews. Over time, the average rating score decreased from 4.7 to 4.1, and waiting and duration became more salient keywords. Overall, vaccination centers appear to be positively perceived, yet users became more critical over the one-year period of the vaccination campaign observed. The study shows that online reviews can provide real-time insights into newly set-up infrastructures, and policymakers should consider their use to monitor the population’s experience over time.

**Keywords:** mass vaccination centers; national vaccination campaign; vaccine uptake;
patient satisfaction; patient experience; health services design; pandemic response; online reviews; natural language processing; machine learning

1. Introduction

In the wake of large-scale COVID-19 pandemic vaccination campaigns starting in December 2020, numerous countries across the globe opted to use vaccination centers as one pillar of their vaccination campaign [1]. A vaccination center is “a location, normally used for non-healthcare activities, set up for high-volume and high-speed vaccinations during infectious disease emergencies” [2]. In Germany, as in many other high-income countries, mass vaccination centers were a novel construct, as vaccinations are usually provided in outpatient practices.

Positive user experience of healthcare is an inherent goal by itself [3]. Well-designed vaccination services can also contribute to higher vaccine uptake [4]: they can increase perceived acceptability and lower adherence barriers [5,6], satisfied users appear more likely to comply with the vaccination schedule (i.e., get a follow-up shot) [7], and recounted personal experiences could serve as a cue to action for others, according to the health belief model [8,9]. In the context of a pandemic, with the challenge of getting most of the population vaccinated [10], user experience can thus be a crucial driver for a successful mass vaccination campaign.

Patient online expressions (e.g., via tweets or review platforms such as Google) have grown exponentially in the past decade [11] and have proven to provide valuable insights into patient experience and satisfaction [12–19] and also towards vaccines and vaccinations, in the context of measles or more recently COVID-19 [20–29]. Vaccination experiences or temporary pandemic response structures within or outside of the context of COVID-19, however, have not yet been studied using patient online expressions. Overall, studies on user experiences of mass vaccination centers are still limited.

Using Google Maps online reviews, we therefore aimed to understand user experience of vaccination centers in Germany. We further aimed to identify key determinants of their experience, which may assist center operators, healthcare providers, and policymakers in optimizing vaccination center design and vaccination processes. We found that the vaccination centers included in our study were perceived positively overall, yet users became more critical as the vaccination campaigns lasted, in particular with regards to efficiency. Policymakers should carefully monitor user experience and its determinants over time. Online reviews can help provide real-time insights for this.

2. Materials and Methods

Online reviews of vaccination centers in Berlin, Germany, were analyzed using natural language processing (NLP). Review texts were analyzed overall, by sentiment, and by time period. A framework for vaccination user experience was developed. The study followed the STrengthening the Reporting of OBservational studies in Epidemiology (STROBE) [30] reporting guideline for cross-sectional studies. It was deemed exempt from informed consent as it used publicly available data.

2.1. Data Collection

The German capital Berlin was chosen as a study site since online reviews were available for all its six vaccination centers. This provided a large sample and allowed the team to study an entire federal state. Furthermore, Berlin hosts a diverse population, promising heterogeneous reviewers. Vaccination centers accounted for approximately 40% of all COVID vaccinations administered in Berlin between December 2020 and December 2021 [31]. The supplemental material provides more details (Appendix A; [32–41]).

Google Maps reviews for all six COVID vaccination centers were retrieved for 12 consecutive months, from 27 December 2020 (start of the vaccination campaign) to 26 December 2021. A dataset containing 3797 online reviews was extracted from publicly available Google Maps reviews sites (Appendix B). Online reviews are provided voluntarily and contain, at a minimum, a star rating ranging from one star to five stars, where one star is the lowest score and five stars is the highest score. In addition, reviews can contain a free text comment. Among other items, the dataset extracted included the following: a review rating ranging from one star to five stars; a timestamp detailing date and time when the review was submitted; a review text. Reviews dating before the opening and after the closing of a site were excluded. For one site (a festival arena) that did not have a dedicated review sub-site, reviews that clearly related to the event location rather than to the vaccination center were removed. Non-English reviews were translated to English using Google Translate [42].

2.2. Analysis

1. *Key Themes*

An analysis of key themes and sentiments was conducted in Python 3.9.9 [43] on all reviews containing text using validated machine learning algorithms: topic modeling with Latent Dirichlet Allocation (LDA) and keyword extraction with Bidirectional Encoder Representations from Transformers (BERT).

LDA is a probabilistic topic modeling algorithm. It extracts word clusters called “topics” that are distributed across texts and connected as latent constructs based on their relative frequency and location [44]. Review texts were pre-processed for LDA using the *NLTK* Python library [45]. The list of stop words, i.e., words to exclude, was amended to also exclude words such as “COVID” and “vaccine” that did not add meaning (Figure S1). LDA was then run on the entire text using the *Gensim* Python library [46]. To refine the model parameters and ensure the topics are interpretable and meaningful, the model was evaluated based on topic coherence (C_v measure) and the intertopic distance map. The *C_v* coherence score was calculated for 2 to 15 topics to determine the optimal number of topics. The Dirichlet hyperparameters α and η were set to default [46]. For topic labeling, each topic’s salient terms were examined based on term frequency (λ = 1.0) and relevance (λ = 0.6) as Sievert and Shirley suggested [47], and sample reviews were inspected. The weight parameter λ ranged from 0 to 1. A smaller λ indicates rare but exclusive terms for the topic, whereas a larger λ features frequent but not necessarily exclusive words [25].

For validation and a more granular look into the reviews overall and sub-groups, automated keyword extraction was used. Keyword extraction identifies single salient words based on semantics. The recent NLP model keyBERT [48–50] was used, which can identify more than one keyword in each text. For example, in “Super organized and very friendly staff.”, keyBERT identified “staff”, “organized”, and “friendly” as keywords. Text pre-processing is not needed, but as with topic modeling, additional stop words were added. Synonyms were then aggregated through lemmatization and manual review (e.g., “employees”, “staff”, and “helper” were grouped to “staff”; “appointment” and “appointments” to “appointment”; Table S1). To ensure the relevance of the results, only words with high frequency were considered in the analysis. Keywords identified in at least 1% (28/2740) of reviews analyzed were then grouped and sorted to a user experience framework (see Section 3.2.). Sub-set analyses were conducted by sentiment and time period. All extractions were validated by one human investigator on a random sub-sample of 30 reviews.

1. *Key Interactions*

A vaccination center user experience framework was developed to summarize the findings and draw actionable conclusions (Appendix C). User experience was defined as the combination of a vaccine recipient’s user journey through the vaccination center and the key determinants of their experience at the vaccination center. User journey phases were defined according to the German Ministry of Health’s handbook for vaccination centers [37]. Key determinants of user experience, such as accessibility, hygiene, and duration, were identified through policy documents on vaccination center planning [37] and case studies of vaccination centers [38,39], as well as existing Patient Reported Experience Measures (PREMs) frameworks [40,41]. These determinants were clustered into 3 themes: staff, process/management, and location. Cost was omitted from the framework, as COVID-19 vaccinations were freely available in Germany.

1. *Timeline*

To understand user responses during different phases of the vaccination campaign, seven time periods were defined based on population eligibility and the setup of alternative vaccination structures over time (Figure 1). In the beginning, vaccinations were solely offered in vaccination centers based on appointments and priority groups. As the campaign progressed, eligible groups were gradually expanded, and other structures (e.g., outpatient practices) could offer vaccines. From June 2021 (period 4) onwards, the prioritization of certain population groups was abandoned, and vaccinations were offered to anyone above the age of 12 in vaccination centers. From July 2021 (period 5) onwards, walk-ins without appointment were introduced [36], and the first vaccination centers closed. September 2021 (period 6) marked the start of the booster campaign for elderly, and November 2021 (period 7) marked the start of the booster campaign for all.

**Figure 1.** Overview of time periods for analysis from December 2020 to December 2021.

1. *Satisfaction*

To understand which themes elicit satisfaction or dissatisfaction, keyword analysis was conducted separately on positive and negative reviews. Users directly expressed their sentiment through review ratings ranging from one to five stars. To ensure that the review rating correlated with the review text, a Pearson correlation coefficient between the sentiment of the review text and the star rating was calculated using *NLTK* and *SciPy* Python libraries [45,51]. Since the Pearson coefficient showed a statistically significant correlation, the rating score was used for further analysis. A review was deemed positive if it received four or five stars, neutral if it received three stars, and negative if it received one or two stars. Users leaving a positive review were assumed to be satisfied, and those leaving a negative review were considered dissatisfied [14].

3. Results

After exclusion, the final dataset contained 3647 reviews. Of these, 907 (25%) solely contained a rating text, and 2740 (75%) contained rating and text (Figure 2A). The average length of a review text was 33 words in English, with a minimum of 1 and a maximum of 544 words.


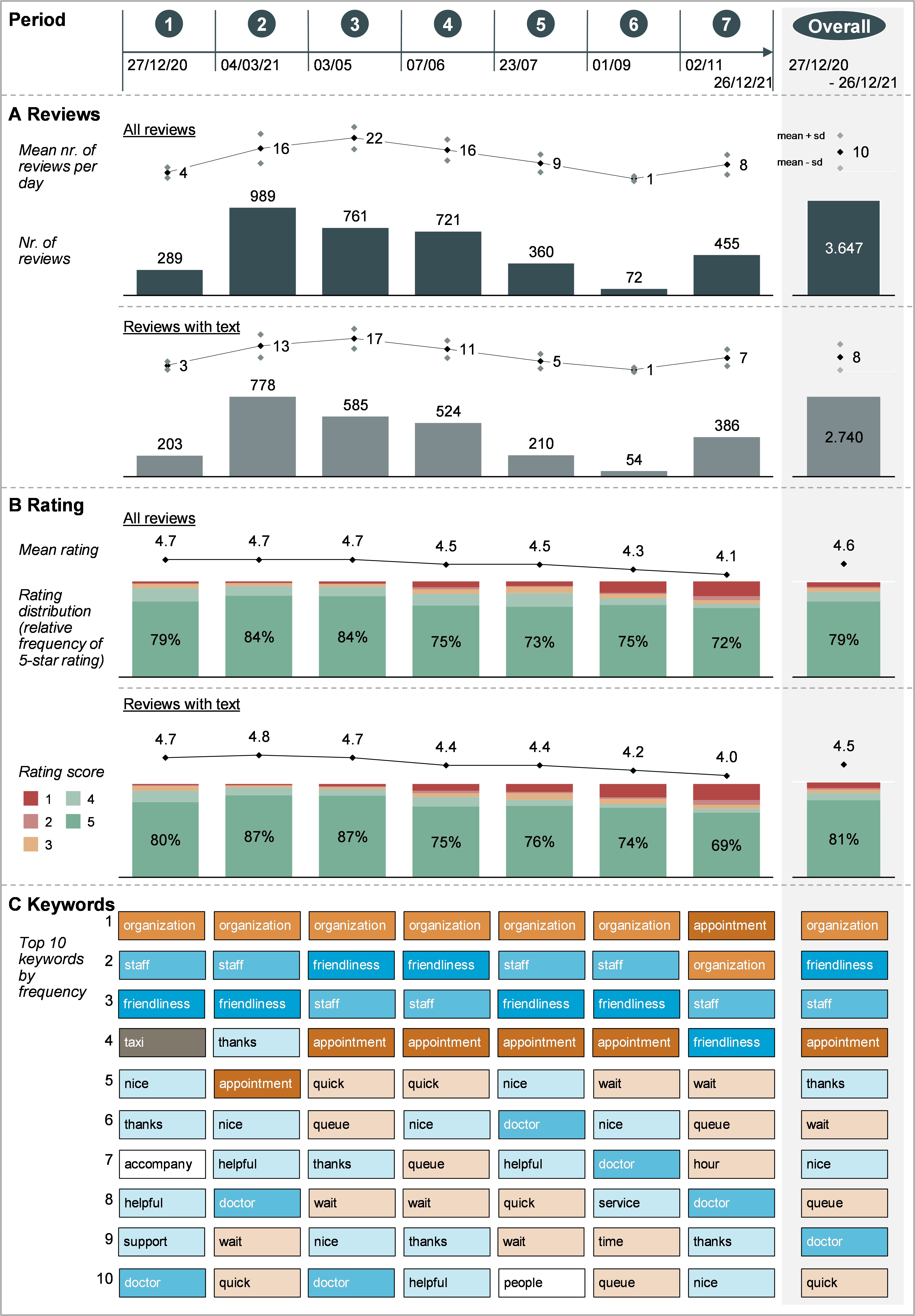


**Figure 2.** Number of reviews (**A**), review rating (**B**), and keywords (**C**) overall and over time.

3.1. Key Themes

Topic modeling with LDA identified 5 key topics. Keyword extraction identified 47 keywords.

For topic modeling, five topics received the highest coherence score (0.486). The five topics (Figure 3, Table S2) covered the vaccination process, location, and staff. Topics 1 and 4 contained comparatively narrow terms. Topics 2, 3, and 5 were more convoluted. Topic 1 was related to scheduling and wait time, and Topic 4 was related to staff friendliness and overall organization. Topic 2 contained terms related to efficiency and duration (“quick”, “start”, and “finish”), vaccination effects, and personal protective equipment (“mask” and “ffp”). An inspection showed that some users reported the effects they experienced shortly after their vaccination, e.g., “[...] I tolerated the vaccine without side effects, only the shoulder hurt a little, like sore muscles. [...]”. Reviews mentioning masks discussed subjects ranging from complaints about security staff not wearing masks to the provision of masks at the centers. Topic 3 broadly dealt with the location, specifically its accessibility (“parking”, “welcome”, and “entrance”), uniqueness (“effort”, “event”, and “concert”), and the intuitiveness of the process exemplified through the term “uncomplicated”. Several reviewers commented on the vaccination being a special event, but also on the fact that a location housed events before becoming a vaccination center. Topic 5 again encompassed site access (“bus”, “shuttle”, and “entrance”), but also registration and documentation (“certificate”, “code”, and “digital”). Inspecting the marginal topic distribution (Figure 3A), the two most salient topics were 1 and 4. Topic 5, regarding accessibility and documentation, was also quite prominent.

**Figure 3.** Results from topic modeling. (**A**) Intertopic distance map of five optimally latent topics and (**B**) top 10 terms per topic by frequency and relevance. In the intertropic distance map, each circle represents one topic. The circle size represents the relative number of terms that belong to the topic. The distance between circles represents the relative similarity and connectedness of topics. Topic circles that are closer to each other have more terms in common. For the top 10 terms per topic, the most frequent terms within a topic are shown at λ-value 1, and the top terms combining frequency and relevance are shown at λ-value 0.6. Relevance reflects the level at which a term exclusively belongs to a single topic. The λ-values 1 and 0.6 are suggested by the prior literature to analyze topics (see Section 2.2).

Keyword extraction identified 47 keywords overall that featured in at least 1% (28/2740) of reviews containing text. The top 30 keywords by frequency are displayed in Figure 4. Four terms were featured in more than 10% (274/2740) of reviews: “organization” (45%, 1227/2740), “friendliness” (34%, 940/2740), “staff” (34%, 940/2740), and “appointment” (17%, 455/2740). Overall, most terms were related to staff and process, few to location, and some to undefined dimensions, such as “people” (5%, 132/2740) and “user” (2%, 48/2740) (Figure S2).


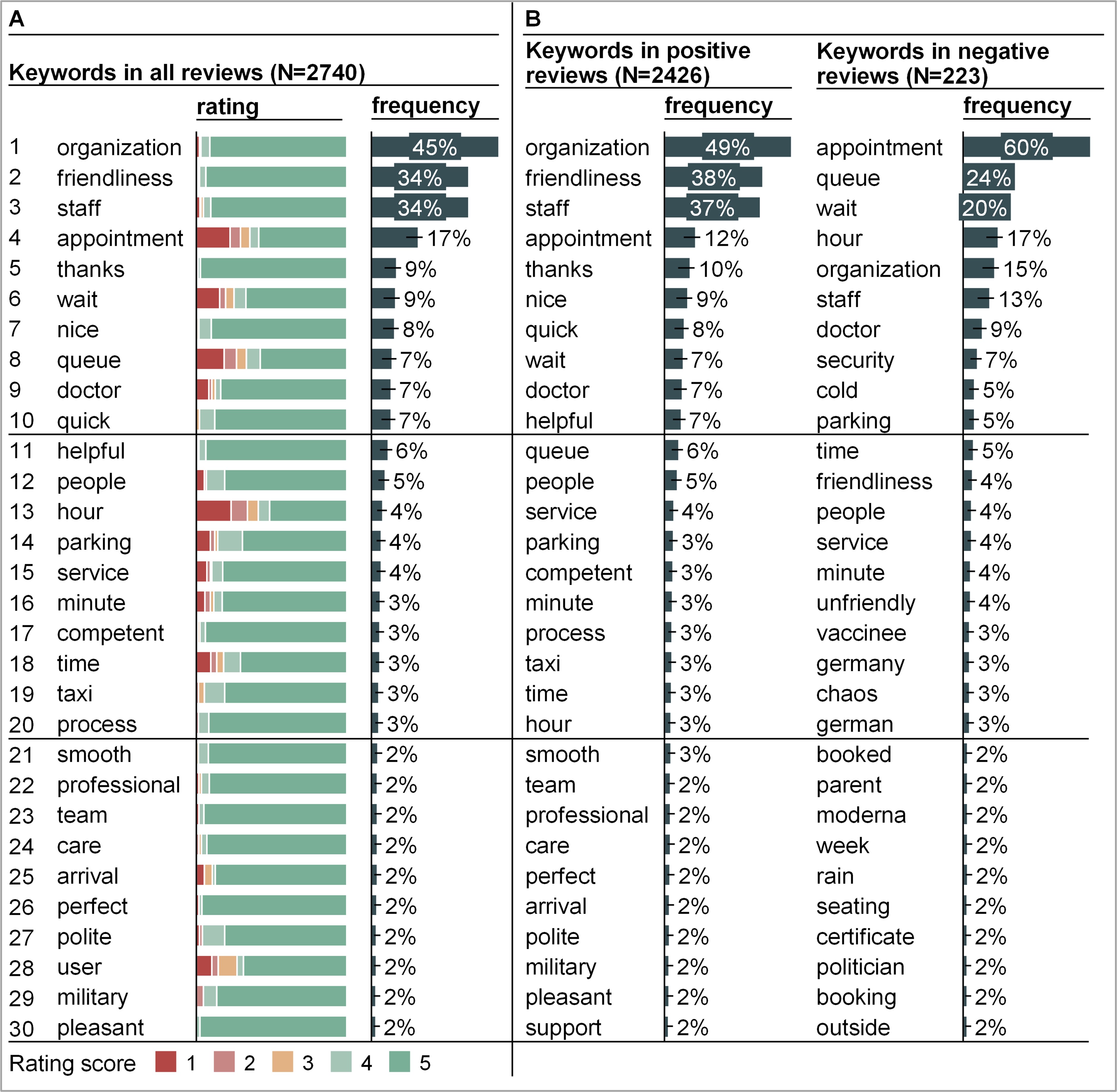


**Figure 4.** Top 30 keywords identified through keyword extraction. (**A**) Top 30 keywords featured across all reviews (N = 2740), sorted by frequency. (**B**) Top 30 keywords featured in positive reviews (four- or five-star rating, N = 2426) and negative reviews (one- or two-star rating, N = 223), sorted by frequency.

3.2. Key Interactions

The 47 keywords were mapped against the user experience framework. Overall, more keywords were related to key determinants of the experience rather than moments in the vaccination center user journey (Figure 5). All three key determinant themes (staff, process/management, and location) were mentioned in reviews. Terms related to overall organization (52%, 1426/2740) and friendliness/responsiveness (49%, 1331/2740) were more frequent than those related to patient flow/wait time (25%, 688/2740), duration/efficiency (18%, 497/2740), and accessibility (12%, 321/2740). Intuitiveness, information/education, and hygiene/infection prevention and control were seldom or not featured.

**Figure 5.** Keywords featured in at least 1% of reviews sorted to the user experience framework. Keywords identified through the keyword extraction were grouped and then mapped against the framework to highlight key interactions and identify recurring themes across reviews. A single review could contain keywords related to several dimensions or phases, e.g., both “organization” and “staff”. A single keyword may also simultaneously be related to an enabler and the journey, e.g., “wait”, which is related to “waiting” in the journey and to “patient flow/wait time” under enabler dimensions. The frequency count of the enabler dimensions or journey phases hence do not add up to the total number of reviews.

There were five key moments in the user journey that were frequently mentioned: scheduling, transit, arrival, waiting, and the vaccination itself. Most keywords were related to the visit itself, while only a few were concerning experiences outside of the vaccination center pre- or post-visit.

3.3. Timeline

The number of reviews varied over time (Figure 2A). In period 2 (when more—also younger—individuals gained access to vaccinations), the number of reviews steeply increased. There was a noticeable drop in reviews after period 5. The top keywords by time period largely overlapped with overall keywords (Figure 2C). Organization and staff were salient in all periods. The three terms “organization”, “staff”, and “friendliness” consistently dominated the top three keywords across all periods until period 7. Other words related to friendliness, e.g., “helpful”, “nice”, and “thanks”, were salient in all periods but became less prevalent over time. Terms related to waiting and duration, such as “wait”, “quick”, “queue”, and particularly the term “appointment”, became gradually more salient from period 2 onwards. “Taxi”, “accompany”, and “support” are only featured among the top 10 keywords in period 1.

3.4. Satisfaction

Overall, 3261 (89%) of the 3647 reviews received a positive rating, 250 (6%) received a negative rating, and 136 (4%) received a neutral rating (Figure 2B). The rating distribution was strongly unimodal, with 2886 (79%) five-star ratings.

Over time, ratings stayed relatively constant with little variance until period 3. In period 4, average ratings started to decrease. Similar to the overall keywords, the keywords identified in positive (N = 2426) and negative (N = 223) reviews containing text were mostly linked to organization, staff, and appointments (Figure 3B).

Positive reviews focused on organization, staff, friendliness, and competence. Negative reviews featured several keywords related to waiting and duration. Specific staff were salient in negative reviews, namely “doctor” (9%, 20/223) and “security” (7%, 15/223) (Figure 3B). An inspection of reviews illustrates this: “outside the security is unfriendly and self-absorbed, inside everyone is very nice. [...]”. The military, which supported center operations, was not featured in negative reviews but appeared in the positive reviews. Furthermore, negative reviews contained several keywords not salient in the reviews overall. These were linked to the weather (“rain” (2%, 5/223), “cold” (5%, 12/223), and “outside” (2%, 4/223)), accessibility (“parking” (5%, 12/223)), general “chaos” (3%, 7/223) and “german” (3%, 7/223). For example, one review reads: “[...] I stood outside in the cold for 1 h at 2 degrees and wind. [...]”.

4. Discussion

4.1. Principal Results

This study had three principal findings. First, the overall reception of vaccination centers was strongly positive. Second, the most important themes identified in the online reviews were wait time, overall organization, and friendliness, while the most important moments in the user journey were scheduling, transit, arrival, waiting, and vaccination. Third, efficiency/duration and wait time/patient flow were leading drivers for dissatisfaction, and their prevalence increased over the vaccination campaign as satisfaction overall decreased.

While many online review-based studies in healthcare [11,24,28,52] and consumer research [52–54] observed a positive rating tendency, online reviews of vaccination centers were extremely skewed compared to those of studies in other settings [11]. Two survey-based studies of COVID-19 vaccination experience in Saudi Arabia and Saxony (Germany) [55,56] found similarly high overall satisfaction scores of above 90% for vaccination centers. Aside from actual experiences or self-selection, this positive skew could be linked to the gratitude and hope associated with the COVID vaccination [57], specifically in the beginning of the vaccination campaign. These feelings could also explain the satisfaction decrease over time; at the start, COVID vaccines were rare, and users were likely more appreciative of the opportunity to get vaccinated, especially early target populations that were more exposed and vulnerable to the virus. As the campaign lasted, the vaccine became more of a commodity. User expectations with regards to the vaccination process probably increased, while recipients became less eager and dependent on vaccines. This seems to fit with the timeline analysis: once the prioritization was abandoned in period 4 and vaccines were offered to anyone aged 12 and above, the rating scores dropped more noticeably (Figure 2B). Additionally, after period 5, only two centers stayed open, and, in period 7, the time between initial and booster vaccination was suddenly decreased, leading to “chaos” [58] around Christmas 2021. Both put pressure on scheduling and impacted accessibility.

Two studies previously evaluated English language tweets on COVID-19 vaccination after the vaccine rollout, irrespective of geographical location, and also found that scheduling and appointments were prevalent themes [25,28]. A systematic review found that important themes in patient online reviews generally included physicians’ demeanor, staff friendliness, time spent with patients, ease of scheduling, wait time, and cost [11]. While staff interaction and appointment management were reflected in the vaccination center reviews, costs were irrelevant in the context of our study. With regards to time spent with patients, vaccination center users seemed more focused on efficiency (i.e., little time spent at the vaccination site) and the competence and friendliness of staff rather than ample exposure time to doctors. A survey-based study comparing vaccination centers and GP offices in Saxony, a more rural German region, found that wait times at mass vaccination centers were lower than they were at GP offices [55].

Topic modeling provided five topics with broader terms that could not always be distinctly sorted to a single determinant, whereas keyword extraction provided keywords more focused on distinct determinants. Both methods identified similar principal themes overall, which increases confidence in the results. Furthermore, the spheres indicating the topic fields in the intertopic distance map for LDA topic modeling (Figure 3) do not overlap, which can be understood as a characteristic of good quality, yet it is noteworthy that keyword extraction did not determine terms related to side effects or documentation (e.g., “certificate” and “code”) as salient. They were featured, however, in Topics 2 and 5 of topic modeling. “Side effects” were also a prevalent topic identified in analyses of tweets related to COVID-19 vaccine rollout [25,28].

Studies cite a range of critical themes in negative online reviews: discordant expectations (education, support, and promises) and sub-optimal communication and quality of care (management, organization, staff, and equipment) [12,60,61]. These were only partly reflected in our findings. As vaccination centers are single-purpose facilities under special circumstances, user priorities appear different from those of traditional healthcare delivery. Huangfu and colleagues [28] also found that appointments played a major role in negative tweets about COVID-19 vaccine rollout. In our study, scheduling, wait time, patient flow, and duration became more salient as the vaccination campaign progressed. In 2020, Volpp and colleagues highlighted the need to reduce these “hassle factors” as crucial for driving COVID vaccine uptake [10]. Dysfunctional scheduling in particular has been a common criticism from the beginning of the German vaccine rollout [59]. Scheduling, which influences wait time, and choice of location, which influences accessibility, lie outside of the vaccination site operators’ control, however. Both are managed by the public administration. To address these critical user experience themes, cross-stakeholder collaboration is necessary.

4.2. Limitations

Our study has three key limitations. First, it is important to note that online reviews are voluntary and predominantly anonymous; they hence suffer from self-selection bias, rely on user sincerity and certain groups appear more prone to providing them, i.e., this limits representativeness. A survey of patients in Germany showed that younger, female, more educated, and chronically ill people were more likely to use patient-review websites [60]. The reviews used in this study lacked information on reviewer characteristics and motives. Furthermore, reviews may not be left by actual service users [61]. A manual examination showed that some reviews were written by companions, e.g., “I went to the vaccination with my 90-year-old mother today. […]”. As the objective was to understand general population response, this does not tarnish the results.

Second, although this study used a large sample covering an entire federal state in Germany over different phases of the COVID-19 vaccination campaign, it studied only one geographical area. Berlin may be particular as vaccination centers were central to the state’s vaccine rollout and some reviews mirror local specificities, e.g., the existence of language barriers. Furthermore, experiences from a well-communicated city-state with a balanced distribution of centers may not translate to rural areas. A study showed that the share of people able to use public transport was higher and the median travel time to vaccination centers in Berlin was lower than they were in other German federal states [62]. It would hence be interesting to see future studies apply the methodology to other geographical areas.

Third, the text quality, specifically related to emojis and translations, was challenging. A total of 258 reviews used emojis. An inclusion was attempted using demoji [63] but produced misleading results in thematic analysis (e.g., light, face, skin, and syringe). Colloquial language, spelling mistakes, and wordplay were not always perfectly translated to English. Some, but not all, translation errors, e.g., “snake” for the German word “Schlange”, which means both queue and snake, could be corrected at the stage of keyword merging. Despite machine learning techniques having made tremendous progress, this highlights some of the currently persisting shortcomings of computer-aided analysis. Improved algorithms for the translation and the analysis of non-English texts and emojis are needed.

To our knowledge, this is the first study to systematically assess vaccination centers from a user’s perspective in Germany using online reviews and the first to assess pandemic response structures using NLP. It lays the foundation for future research in this area and contributes to pandemic response planning. Aside from addressing the limitations above, future research should include a comparison to other vaccination structures, e.g., physicians’ offices, and other single-purpose healthcare structures, e.g., test centers, and vaccination experiences outside of a global pandemic. It should also contrast online reviews with traditional survey results to further gauge their potential.

5. Conclusions

Positive user experience seems a worthwhile investment for decision-makers and operators during a pandemic, where adherence to services and societal cohesion are essential. Overall, the results of the study with regards to vaccination centers as a pandemic mass vaccination structure are encouraging: User receptions of vaccination centers were very positive, and smooth processes and friendly staff were highly valued. As the mass vaccination campaign lasted, however, efficiency and time management needed to be monitored to ensure long-term satisfaction. Online reviews provided useful, free, and real-time feedback. This makes them an attractive, early recognition “armchair epidemiology” tool [64] for evaluating novel structures during crises, where circumstances continuously change. Their use in pandemic response should therefore be strengthened—encouraging user reviews, establishing units in public administration capable of their analysis, and promoting an iterative design mindset.

6. Patents

Not applicable.

**Supplementary Materials:** The following supporting information can be downloaded at [www.mdpi.com/xxx/s1](http://www.mdpi.com/xxx/s1), **Figure S1**: Data pre-processing procedure for LDA topic modeling. **Table S1:** Word grouping for keyword analysis. **Table S2:** Top 30 keywords from topic modeling by frequency and relevance. **Figure S2**: Keywords contained in at least 1% of reviews sorted by key determinant.

**Author Contributions:** Conceptualization, S.D. and F.S.; methodology, S.D., J.K., and M.B.; data curation, S.D. and M.B.; software, M.B.; validation, J.K., M.B., and S.D.; formal analysis, S.D. and M.B.; investigation, S.D. and M.B.; writing—original draft preparation, S.D.; writing—review and editing, F.S., S.D., M.B., and J.K.; visualization, S.D. and M.B.; resources, F.S.; project administration, S.D. All authors have read and agreed to the published version of the manuscript.

**Funding:** This research received no external funding. The APC was funded through the authors’ institution (Charité—Universitätsmedizin Berlin).

**Institutional Review Board Statement:** Not applicable.

**Informed Consent Statement:** Not applicable.

**Data Availability Statement:** Publicly available data was used. See Appendix B for more details.

**Acknowledgments:** Not applicable.

**Conflicts of Interest:** The authors declare no conflicts of interest.

Appendix A


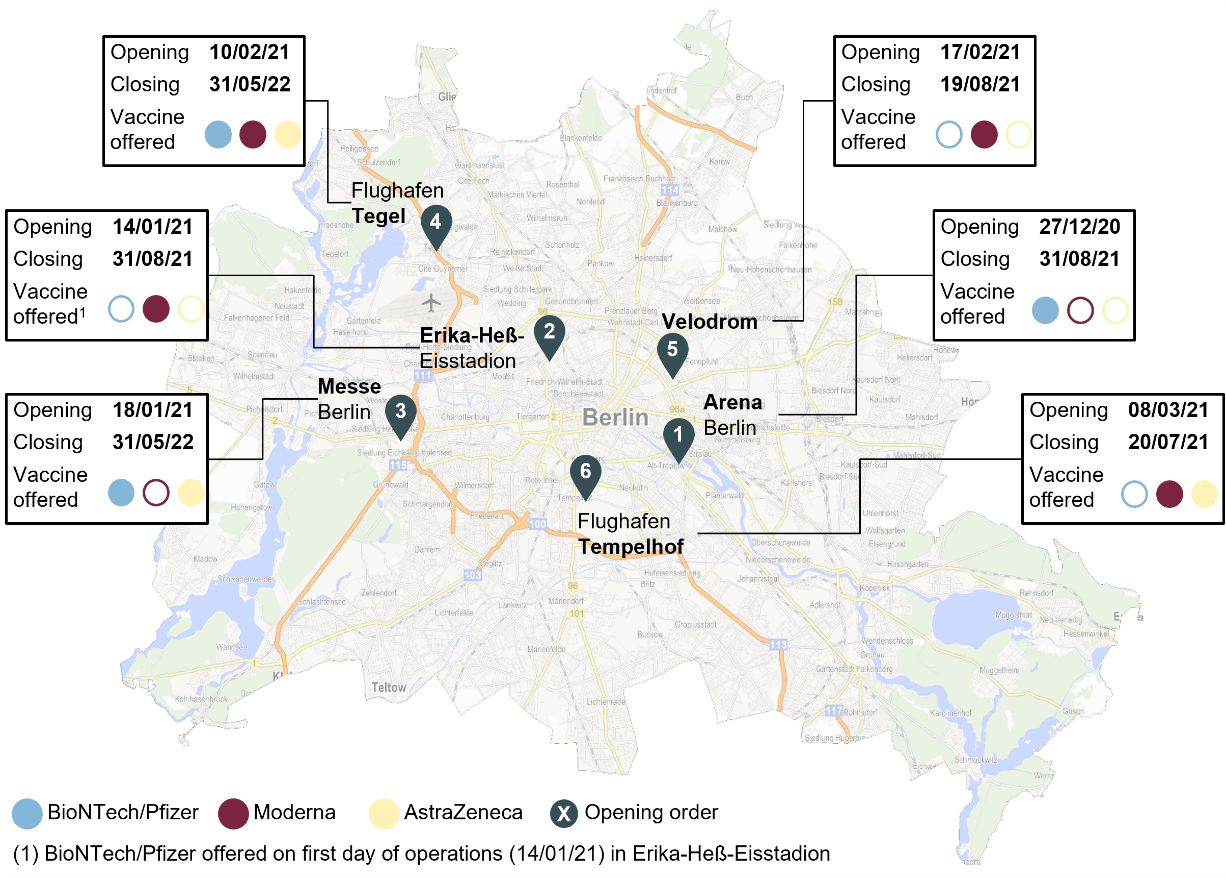


**Figure A1.** Overview of COVID-19 vaccination centers in Berlin.

A total of six mass vaccination centers were set up in Berlin and opened between December 27, 2020, and March 8, 2021. The centers were set up by or on behalf of the federal states and financed through public funds[32]. In Berlin, the centers were operated by several Berlin-based humanitarian aid organizations coordinated through DRK SWB, a non-profit company established by the German Red Cross Berlin, the Association of Statutory Health Insurance Physicians Berlin and the Senate Department for Health, Nursing and Equality. The vaccination centers were spread across the city to ensure reachability from all districts. The vaccination centers were open daily usually between 9AM and 5PM with a few exceptional closures (e.g., two sites closed temporarily due to a suspension of AstraZeneca while side effects were being reviewed[33,34]). A single vaccination center could perform up to 4.000 vaccinations per day[35]. The effective daily volume depended on the number of vaccines available, and the number of appointments booked. Three different vaccines were available at the vaccination centers. In the beginning vaccinations were offered based on appointments and priority group, from July 2021 walk-ins were introduced[36]. As the vaccination campaign progressed and other structures (e.g., outpatient practices and company physicians) got involved, vaccination centers were gradually closed. At the time the study was conducted two vaccination centers were still running.

Appendix B

The raw data was obtained on 27 dec 2021 from Google Maps Reviews public review websites (Table A1) using Outscraper (<https://outscraper.com>). Online reviews for all six vaccination centers in Berlin were obtained for the period 27 dec 2020 00:00:00 to 27 dec 2021 00:00:00. The dataset used for the analysis can be found on github at: <https://github.com/stellaroxanne/User-Reviews-Vaccination-Centers-Berlin> (last accessed 27 Dec 2022).

**Table A1.** Links to Google Maps Online Review sites for the vaccination centers analyzed.

| **Name Vaccination Center** | **Google Maps Online Review Link** |
| --- | --- |
| Arena | <https://www.google.com/maps/place/Arena+Berlin/@52.4964613,13.4543597,15z/data=!4m5!3m4!1s0x0:0x6852fd9350063186!8m2!3d52.4964613!4d13.4543597> (last accessed 27 Dec 2022) |
| Erika-Heß-Eisstadion | <https://www.google.com/maps/place/Impfzentrum+Berlin+-+Erika-He%C3%9F-Eisstadion/@52.537194,13.3674287,17z/data=!3m2!4b1!5s0x47a8518fec26cc1b:0x9d079a3d045872a3!4m5!3m4!1s0x47a8516968c04057:0x759b414ae8c704fc!8m2!3d52.537194!4d13.3696174> (last accessed 27 Dec 2022) |
| Flughafen Tegel | <https://www.google.com/maps/place/Impfzentrum+Berlin+Tegel+Terminal+C/@52.555381,13.293457,17z/data=!3m1!4b1!4m5!3m4!1s0x47a857e0f5b20727:0x27042a6252ee947c!8m2!3d52.555381!4d13.2956457> (last accessed 27 Dec 2022) |
| Flughafen Tempelhof | <https://www.google.com/maps/place/Impfzentrum+Flughafen+Tempelhof/@52.4827589,13.3909385,17z/data=!3m1!4b1!4m5!3m4!1s0x47a84f62dae9d565:0xd5b04abca92add1f!8m2!3d52.4827589!4d13.3931272> (last accessed 27 Dec 2022) |
| Messe | <https://www.google.com/maps/place/Corona-Impfzentrum+Messe+Berlin/@52.5063417,13.2704876,17z/data=!3m1!4b1!4m5!3m4!1s0x47a8571f61b570a7:0x6db977b39837a08a!8m2!3d52.5063417!4d13.2726763> (last accessed 27 Dec 2022) |
| Velodrom | <https://www.google.com/maps/place/Impfzentrum+Berlin+-+Velodrom/@52.5305156,13.4486547,17z/data=!3m1!4b1!4m5!3m4!1s0x47a84fc3a22029cf:0x2e9371eb78584ce8!8m2!3d52.5305156!4d13.4508434> (last accessed 27 Dec 2022) |

Appendix C

The figure below (Figure C1) shows the final framework for vaccination center user experience composed of user journey and key determinants.

The user journey follows the path of a vaccination center user from their invitation to get vaccinated up to their follow-up appointment. It is composed of three phases: pre-visit, visit and post-visit. The user journey can also be applied to vaccinations in other settings as it follows a similar overarching path.

The table below (Table C1) shows the different publications on vaccination centers and patient reported outcome and experience measures that were referenced to determine the relevant key determinants for a positive vaccination experience. The key determinants that form part of the final framework can be clustered into three groups: staff, process/management and location.


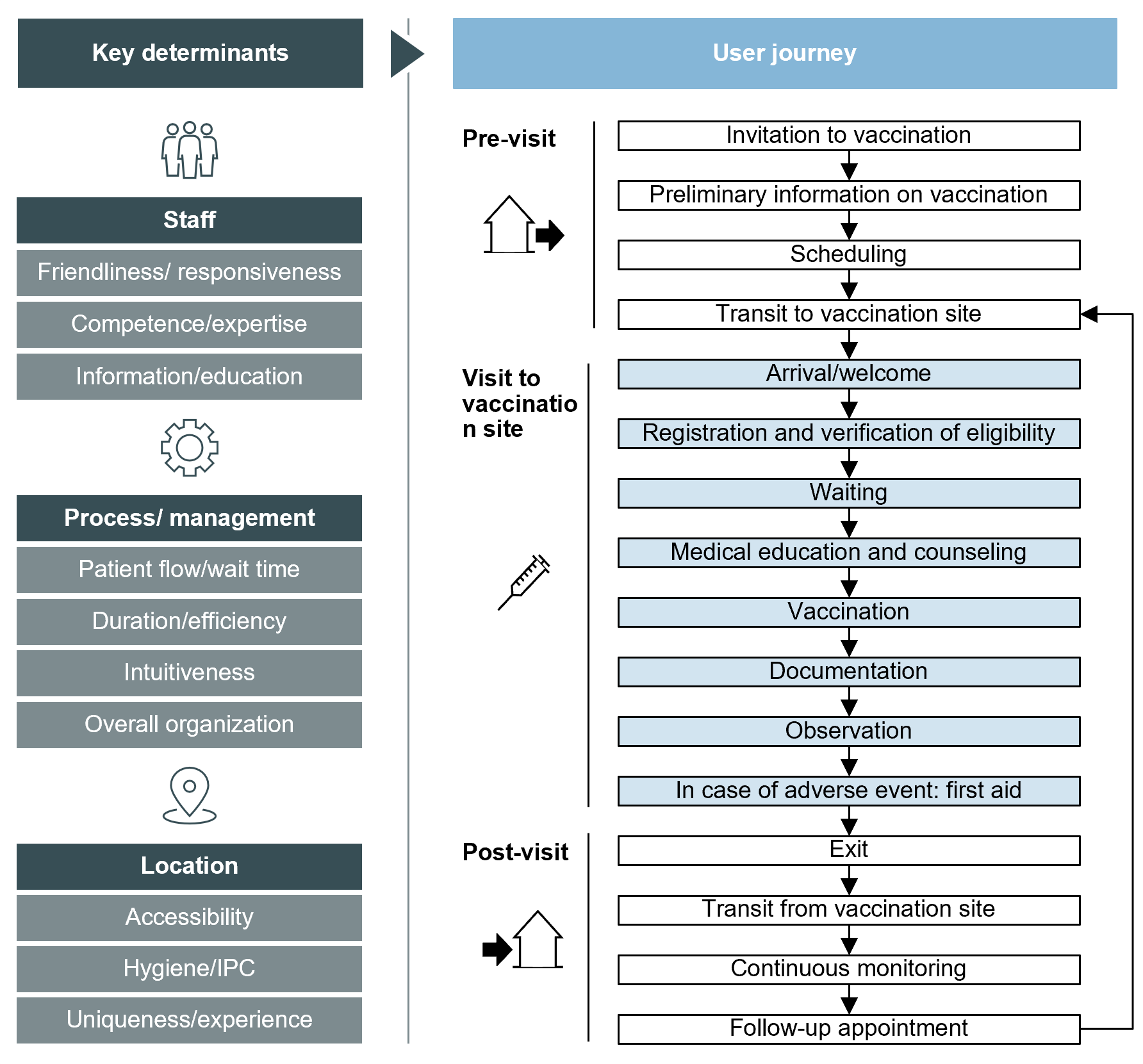


**Figure A2:** Framework for vaccination center user experience including key determinants of user experience and the vaccination center user journey.

**Table 2.** Key determinants of user experience as referred to in publications on vaccination centers or reference publications on patient reported experience measures (PREMs) and patient reported outcome measures (PROMs).

| **Fields** | **Key determinants** | **Final framework** | **MoH Handbook (2020) [37]** | **Golberg et al. (2021) [38]** | **Goralnick et al. (2021)[39]** | **OECD (2018)[40]** | **Bertelsmann (2010)[41]** |
| --- | --- | --- | --- | --- | --- | --- | --- |
| **Staff** | Friendliness /  responsiveness | **x** |  | x |  | x | x |
|  | Competence /  expertise | **x** | x |  |  |  | x |
|  | Information /  education | **x** | x |  |  | x | x |
| **Process /**  **Management** | Patient flow /  wait time | **x** | x | x |  | x | x |
|  | Duration /  efficiency | **x** |  |  |  | x |  |
|  | Intuitiveness | **x** | x | x |  | x | x |
|  | Overall organization | **x** | x | x |  |  |  |
| **Location** | Accessibility | **x** | x | x | x |  | x |
|  | Hygiene/ IPC | **x** | x | x |  |  | x |
|  | Uniqueness | **x** |  | x |  |  |  |
|  | Reputation | **-** |  |  |  |  |  |
| **Patient choice** | Vaccine availability | **-** |  |  |  |  |  |
|  | Cost | **-** |  |  | x | x |  |

References

1. Hasan, T.; Beardsley, J.; Marais, B.; Nguyen, T.; Fox, G. The Implementation of Mass-Vaccination against SARS-CoV-2: A Systematic Review of Existing Strategies and Guidelines. *Vaccines* **2021**, *9*, 326. https://doi.org/10.3390/vaccines9040326.
2. Gianfredi, V.; Pennisi, F.; Lume, A.; Ricciardi, G.; Minerva, M.; Riccò, M.; Odone, A.; Signorelli, C. Challenges and Opportunities of Mass Vaccination Centers in COVID-19 Times: A Rapid Review of Literature. *Vaccines* **2021**, *9*, 574. https://doi.org/10.3390/vaccines9060574.
3. Hostettler, S.; Kraft, E.; Bosshard, C. Patient-reported outcome measures: Die Patientensicht zählt. Grundlagenpapier der DDQ/SAQM. *Schweiz Ärzteztg* **2018**, *99*, 1348–1352. https://doi.org/10.4414/saez.2018.17187.
4. Kaneko, M.; Aoki, T.; Goto, R.; Ozone, S.; Haruta, J. Better Patient Experience is Associated with Better Vaccine Uptake in Older Adults: Multicentered Cross-sectional Study. *J. Gen. Intern. Med.* **2020**, *35*, 3485–3491. https://doi.org/10.1007/s11606-020-06187-1.
5. Betsch, C.; Schmid, P.; Korn, L.; Steinmeyer, L.; Heinemeier, D.; Eitze, S.; Küpke, N.K.; Böhm, R. Impfverhalten psychologisch erklären, messen und verändern. *Bundesgesundheitsblatt—Gesundh.—Gesundh.* **2019**, *62*, 400–409. https://doi.org/10.1007/s00103-019-02900-6.
6. Penchansky, R.; Thomas, J.W. The concept of access: Definition and relationship to consumer satisfaction. *Med. Care* **1981**, *19*, 127–140. https://doi.org/10.1097/00005650-198102000-00001.
7. Oliver, R.L. A Cognitive Model of the Antecedents and Consequences of Satisfaction Decisions. *J. Mark. Res.* **1980**, *17*, 460. https://doi.org/10.2307/3150499.
8. Becker, M.H.; Maiman, L.A. Sociobehavioral Determinants of Compliance with Health and Medical Care Recommendations. *Med. Care* **1975**, *13*, 10–24. https://doi.org/10.1097/00005650-197501000-00002.
9. van Velthoven, M.H.; Atherton, H.; Powell, J. A cross sectional survey of the UK public to understand use of online ratings and reviews of health services. *Patient Educ. Couns.* **2018**, *101*, 1690–1696. https://doi.org/10.1016/j.pec.2018.04.001.
10. Volpp, K.G.; Loewenstein, G.; Buttenheim, A.M. Behaviorally Informed Strategies for a National COVID-19 Vaccine Promotion Program. *JAMA* **2020**, *325*, 125–126. https://doi.org/10.1001/jama.2020.24036.
11. Hong, Y.A.; Liang, C.; Radcliff, T.A.; Wigfall, L.T.; Street, R.L. What Do Patients Say About Doctors Online? A Systematic Review of Studies on Patient Online Reviews. *J. Med. Internet Res.* **2019**, *21*, e12521. https://doi.org/10.2196/12521.
12. Brereton, E.J.; Matlock, D.D.; Fitzgerald, M.; Venechuk, G.; Knoepke, C.; Allen, L.A.; Tate, C.E. Content Analysis of Negative Online Reviews of Hospice Agencies in the United States. *JAMA Netw. Open* **2020**, *3*, e1921130–e1921130. https://doi.org/10.1001/jamanetworkopen.2019.21130.
13. Gupta, R.; Alraqiq, H.M. Patient experiences at dental school clinics: A comparative analysis of online reviews. *J. Dent. Educ.* **2022**, *86*, 77–87. https://doi.org/10.1002/jdd.12782.
14. Dorfman, R.G.; Purnell, C.; Qiu, C.; Ellis, M.; Basu, C.B.; Kim, J.Y.S. Happy and Unhappy Patients. *Plast. Reconstr. Surg.* **2018**, *141*, 663e–673e. https://doi.org/10.1097/prs.0000000000004268.
15. Tran, N.N.; Lee, J.; Zhang, Q.; Bidmon, S. Online Reviews as Health Data: Examining the Association Between Availability of Health Care Services and Patient Star Ratings Exemplified by the Yelp Academic Dataset. *JMIR Public Health Surveill.* **2017**, *3*, e43. https://doi.org/10.2196/publichealth.7001.
16. Bardach, N.S.; Lyndon, A.; Asteria-Peñaloza, R.; Goldman, L.E.; Lin, G.A.; Dudley, R.A. From the closest observers of patient care: A thematic analysis of online narrative reviews of hospitals. *BMJ Qual. Saf.* **2016**, *25*, 889–897. https://doi.org/10.1136/bmjqs-2015-004515.
17. Emmert, M.; Halling, F.; Meier, F.; Bidmon, S.; Schaefer, C. Evaluations of Dentists on a German Physician Rating Website: An Analysis of the Ratings. *J. Med. Internet Res.* **2015**, *17*, e15. https://doi.org/10.2196/jmir.3830.
18. Smith, R.J.; Lipoff, J.B. Evaluation of Dermatology Practice Online Reviews. *JAMA Dermatol.* **2016**, *152*, 153–157. https://doi.org/10.1001/jamadermatol.2015.3950.
19. Al-Ahdal, T.; Coker, D.; Awad, H.; Reda, A.; Żuratyński, P.; Khailaie, S. Improving Public Health Policy by Comparing the Public Response during the Start of COVID-19 and Monkeypox on Twitter in Germany: A Mixed Methods Study. *Vaccines* **2022**, *10*, 1985. https://doi.org/10.3390/vaccines10121985.
20. Raghupathi, V.; Ren, J.; Raghupathi, W. Studying Public Perception about Vaccination: A Sentiment Analysis of Tweets. *Int. J. Environ. Res. Public Health* **2020**, *17*, 3464. https://doi.org/10.3390/ijerph17103464.
21. Wu, W.; Lyu, H.; Luo, J. Characterizing Discourse about COVID-19 Vaccines: A Reddit Version of the Pandemic Story. *Health Data Sci.* **2021**, *2021*, 1–11. https://doi.org/10.34133/2021/9837856.
22. Melton, C.A.; Olusanya, O.A.; Ammar, N.; Shaban-Nejad, A. Public sentiment analysis and topic modeling regarding COVID-19 vaccines on the Reddit social media platform: A call to action for strengthening vaccine confidence. *J. Infect. Public Health* **2021**, *14*, 1505–1512. https://doi.org/10.1016/j.jiph.2021.08.010.
23. Taeb, M.; Chi, H.; Yan, J. Applying Machine Learning to Analyze Anti-Vaccination on Tweets. In Proceedings of the 2021 IEEE International Conference on Big Data (Big Data), Orlando, FL, USA, 15–18 December 2021; pp. 4426–4430. https://doi.org/10.1109/bigdata52589.2021.9671647.
24. Lyu, J.C.; Le Han, E.; Luli, G.K. COVID-19 Vaccine–Related Discussion on Twitter: Topic Modeling and Sentiment Analysis. *J. Med. Internet Res.* **2021**, *23*, e24435. https://doi.org/10.2196/24435.
25. Zhang, J.; Wang, Y.; Shi, M.; Wang, X. Factors Driving the Popularity and Virality of COVID-19 Vaccine Discourse on Twitter: Text Mining and Data Visualization Study. *JMIR Public Health Surveill.* **2021**, *7*, e32814. https://doi.org/10.2196/32814.
26. Yin, F.; Wu, Z.; Xia, X.; Ji, M.; Wang, Y.; Hu, Z. Unfolding the Determinants of COVID-19 Vaccine Acceptance in China. *J. Med. Internet Res.* **2021**, *23*, e26089. https://doi.org/10.2196/26089.
27. Sarirete, A. Sentiment analysis tracking of COVID-19 vaccine through tweets. *J. Ambient. Intell. Humaniz. Comput.* **2022**, 1–9. https://doi.org/10.1007/s12652-022-03805-0.
28. Huangfu, L.; Mo, Y.; Zhang, P.; Zeng, D.D.; He, S. COVID-19 Vaccine Tweets After Vaccine Rollout: Sentiment–Based Topic Modeling. *J. Med. Internet Res.* **2022**, *24*, e31726. https://doi.org/10.2196/31726.
29. Nguyen, A.; Catalan-Matamoros, D. Anti-Vaccine Discourse on Social Media: An Exploratory Audit of Negative Tweets about Vaccines and Their Posters. *Vaccines* **2022**, *10*, 2067. https://doi.org/10.3390/vaccines10122067.
30. Von Elm, E.; Altman, D.G.; Egger, M.; Pocock, S.J.; Gøtzsche, P.C.; Vandenbroucke, J.P.; Initiative, S. The Strengthening the Reporting of Observational Studies in Epidemiology (STROBE) statement: Guidelines for reporting observational studies. *Lancet* **2007**, *370*, 1453–1457. https://doi.org/10.1016/s0140-6736(07)61602-x.
31. Robert Koch-Institut. *Wöchentlicher Lagebericht des RKI zur Coronavirus-Krankheit Wochenbericht vom 13.01.2022*; Robert Koch-Institut: Berlin, Germany.
32. Bundesministerium für Gesundheit. *Verordnung zum Anspruch auf Schutzimpfung gegen das Coronavirus SARS-CoV-2 (Coronavirus-Impfverordnung—CoronaImpfV)*; Bundesministerium für Gesundheit: Berlin, Germany, 2021.
33. Impfbetrieb in Tegel und Tempelhof Startet am Freitag wieder. Berlin.de—Das offizielle Hauptstadtportal 2021. Available online: <https://www.berlin.de/aktuelles/berlin/6479694-958092-impfbetrieb-in-tegel-und-tempelhof-start.html> (accessed on).
34. Senatsverwaltung für Gesundheit, Pflege und Gleichstellung (2016–2021). Berlin setzt Impfen mit AstraZeneca aus. Berlin: 2021. Available online: https://www.berlin.de/sen/archiv/gpg-2016-2021/2021/pressemitteilung.1064782.php (accessed on 12 April 2022).
35. Bauer, C. Corona in Berlin: Impfzentrum auf dem Messegelände bleibt bis Februar auf. Berl. Morgenpost. 2021. Available online: https://www.morgenpost.de/bezirke/charlottenburg-wilmersdorf/article233947755/Impfzentrum-an-der-Messe-bleibt.html (accessed on 12 April 2022).
36. Senatsverwaltung für Gesundheit, Pflege und Gleichstellung (2016–2021). Impfen ohne Termin jetzt in allen Berliner Impfzentren möglich—zusätzlich zu Moderna auch Biontech. Berlin: : Senatsverwaltung für Gesundheit, Pflege und Gleichstellung (2016–2021). 2021. Available online: <https://www.berlin.de/sen/archiv/gpg-2016-2021/2021/pressemitteilung.1111392.php> (accessed on 27 Dec 2022).
37. German Federal Ministry of Health. *Empfehlungen für die Organisation und Durchführung von Impfungen gegen SARS-CoV-2 in Impfzentren und Mit Mobilen Teams*; German Federal Ministry of Health: Berlin, Germany, 2020.
38. Goldberg, S.A.; Callaway, D.; Resnick-Ault, D.; Mandavia, S.; Martinez, R.; Bass, M.; Goralnick, E. Critical Concepts for COVID-19 Mass Vaccination Site Operations. *Disaster Med. Public Health Prep.* **2021**, 1–7. https://doi.org/10.1017/dmp.2021.319.
39. Goralnick, E.; Kaufmann, C.; Gawande, A.A. Mass-Vaccination Sites—An Essential Innovation to Curb the Covid-19 Pandemic. *New Engl. J. Med.* **2021**, *384*, e67. https://doi.org/10.1056/nejmp2102535.
40. Fujisawa, R.; Klazinga, N. OECD Health Working Papers No. 102—Measuring Patient Experiences (PREMs): Progress made by the OECD and its member countries between 2006 and 2016. In *OECD Health Working Papers*; Organisation for Economic Cooperation and Development (OECD)—Directorate for Employment, Labour and Social Affairs: Paris, France, 2018; p.62.
41. Gehrlachm, C.; Altenhöner, T.; Schwappach, D.L.B. Der Patients’ Experience Questionnaire Patientenerfahrungen Vergleichbar machen. 2010. Available online: https://nbn-resolving.org/urn:nbn:de:101:1-201009078063 (accessed on 19 April 2022).
42. Google Translate. Available online: <https://translate.google.com/> (accessed on 30 Dec 2021).
43. Welcome to Python.org. Available online: https://www.python.org/ (accessed on 3 April 2022).
44. Lee, C.K.H. How guest-host interactions affect consumer experiences in the sharing economy: New evidence from a configurational analysis based on consumer reviews. *Decis. Support Syst.* **2021**, *152*, 113634. https://doi.org/10.1016/j.dss.2021.113634.
45. Bird, S.; Klein, E.; Loper, E. *Natural Language Processing with Python: Analyzing Text with the Natural Language Toolkit*; O’Reilly Media, Inc.: Sebastopol, CA, USA, 2009.
46. Rehurek, R.; Sojka, P. Gensim–python framework for vector space modelling. *NLP Cent. Fac. Inform. Masaryk Univ. Brno Czech Repub.* **2011**, *3*, 2.
47. Sievert, C.; Shirley, K. LDAvis: A method for visualizing and interpreting topics. In Proceedings of the Workshop on Interactive Language Learning, Visualization, and Interfaces, Baltimore, MD, USA, 27 June 2014.
48. Grootendorst, M. Keyword Extraction with BERT. Medium. 2020. Available online: https://towardsdatascience.com/keyword-extraction-with-bert-724efca412ea (accessed on 12 April 2022).
49. Grootendorst, M. KeyBERT: Minimal Keyword Extraction with BERT. 2020. Available online: https://github.com/MaartenGr/KeyBERT (accessed on 27 Dec 2022).
50. D’Agostino, A. Keyword Extraction—A Benchmark of 7 Algorithms in Python. Medium. 2021. Available online: https://towardsdatascience.com/keyword-extraction-a-benchmark-of-7-algorithms-in-python-8a905326d93f (accessed on 12 April 2022).
51. Virtanen, P.; Gommers, R.; Oliphant, T.E.; Haberland, M.; Reddy, T.; Cournapeau, D.; Burovski, E.; Peterson, P.; Weckesser, W.; Bright, J.; et al. SciPy 1.0: Fundamental Algorithms for Scientific Computing in Python. *Nat. Methods* **2020**, *17*, 261–272.
52. Kadry, B.; Chu, L.F.; Kadry, B.; Gammas, D.; Macario, A. Analysis of 4999 Online Physician Ratings Indicates That Most Patients Give Physicians a Favorable Rating. *J. Med. Internet Res.* **2011**, *13*, e95. https://doi.org/10.2196/jmir.1960.
53. Mathayomchan, B.; Taecharungroj, V. “How was your meal?” Examining customer experience using Google maps reviews. *Int. J. Hosp. Manag.* **2020**, *90*, 102641. https://doi.org/10.1016/j.ijhm.2020.102641.
54. Borrego, .; Navarra, M.C. What users say about public libraries: An analysis of Google Maps reviews. *Online Inf. Rev.* **2020**, *45*, 84–98. https://doi.org/10.1108/oir-09-2019-0291.
55. Lee, K.; Yu, C. Assessment of airport service quality: A complementary approach to measure perceived service quality based on Google reviews. *J. Air Transp. Manag.* **2018**, *71*, 28–44. https://doi.org/10.1016/j.jairtraman.2018.05.004.
56. Jentzsch, A.; Geier, A.-K.; Bleckwenn, M.; Schrimpf, A. Differences in Demographics of Vaccinees, Access to, and Satisfaction with SARS-CoV-2 Vaccination Procedures between German General Practices and Mass Vaccination Centers. *Vaccines* **2022**, *10*, 1823. https://doi.org/10.3390/vaccines10111823.
57. Satisfaction with COVID-19 Vaccination Program” by Muhammad Waseem Shahzad, Adel Al-Shabaan et al. Available online: https://pxjournal.org/journal/vol9/iss3/21/ (accessed on 19 December 2022).
58. Kasai, T. COVID-19 Vaccines Offer Hope, Other Prevention Measures Must Continue. WHO West. Pac. 2021. Available online: https://www.who.int/westernpacific/news-room/commentaries/detail-hq/covid-19-vaccines-offer-hope-but-other-prevention-measures-must-continue (accessed on 12 April 2022).
59. Steffen, T. Boosterimpfung: Impfchaos, Schon Wieder. Zeit. 2021. Available online: https://www.zeit.de/politik/deutschland/2021-12/boosterimpfung-impfstoff-corona-krisenstab (accessed on 12 April 2022).
60. Fairie, P.; Zhang, Z.; D'Souza, A.G.; Walsh, T.; Quan, H.; Santana, M.J. Categorising patient concerns using natural language processing techniques. *BMJ Health Care Inform.* **2021**, *28*, e100274. https://doi.org/10.1136/bmjhci-2020-100274.
61. Stokes, D.C.; Pelullo, A.P.; Mitra, N.; Meisel, Z.F.; South, E.C.; Asch, D.A.; Merchant, R.M. Association Between Crowdsourced Health Care Facility Ratings and Mortality in US Counties. *JAMA Netw. Open* **2021**, *4*, e2127799–e2127799. https://doi.org/10.1001/jamanetworkopen.2021.27799.
62. Bitkom. Bundesbürger fordern digitale Mittel im Kampf gegen Corona. Bitkom Presse. https://www.bitkom.org/Presse/Presseinformation/Bundesbuerger-fordern-digitale-Mittel-im-Kampf-gegen-Corona (accessed on 22 April 2022).
63. Terlutter, R.; Bidmon, S.; Röttl, J. Who Uses Physician-Rating Websites? Differences in Sociodemographic Variables, Psychographic Variables, and Health Status of Users and Nonusers of Physician-Rating Websites. *J. Med. Internet Res.* **2014**, *16*, e97. https://doi.org/10.2196/jmir.3145.
64. Lagu, T.; Hannon, N.S.; Rothberg, M.B.; Lindenauer, P.K. Patients’ Evaluations of Health Care Providers in the Era of Social Networking: An Analysis of Physician-Rating Websites. *J. Gen. Intern. Med.* **2010**, *25*, 942–946. https://doi.org/10.1007/s11606-010-1383-0.
65. Neumeier, S. Accessibility of COVID-19 Vaccination Centers in Germany via Different Means of Transport. *KN—J. Cartogr. Geogr. Inf.* **2022**, *72*, 41–58. https://doi.org/10.1007/s42489-021-00088-x.
66. Salomon, B.; Spaans, J. Demoji. 2021. Available online: <https://github.com/bsolomon1124/demoji> (accessed on 15 Feb 2022).
67. Sommer, A. Using the Internet to Detect Emerging Outbreaks—Armchair Epidemiology. *JAMA Ophthalmol.* **2022**, *140*, 50. https://doi.org/10.1001/jamaophthalmol.2021.4853.

**Disclaimer/Publisher’s Note:** The statements, opinions and data contained in all publications are solely those of the individual author(s) and contributor(s) and not of MDPI and/or the editor(s). MDPI and/or the editor(s) disclaim responsibility for any in-jury to people or property resulting from any ideas, methods, instructions or products referred to in the content.
