# Supplementary material for: How Do Users Respond to Mass Vaccination Centers? A Cross-Sectional Study Using Natural Language Processing on Online Reviews to Explore User Experience and Satisfaction with COVID-19 Vaccination Centers
Source: Vaccines (Basel). 2023 Jan 9;11(1):144. doi: 10.3390/vaccines11010144 (PMC9861127; doi:10.3390/vaccines11010144)
Supplement: Supplementary file 1 [file vaccines-11-00144-s001.zip › S-FIGURES_Vaccines_User Experiences Vaccines Centers_Revision 1.docx]

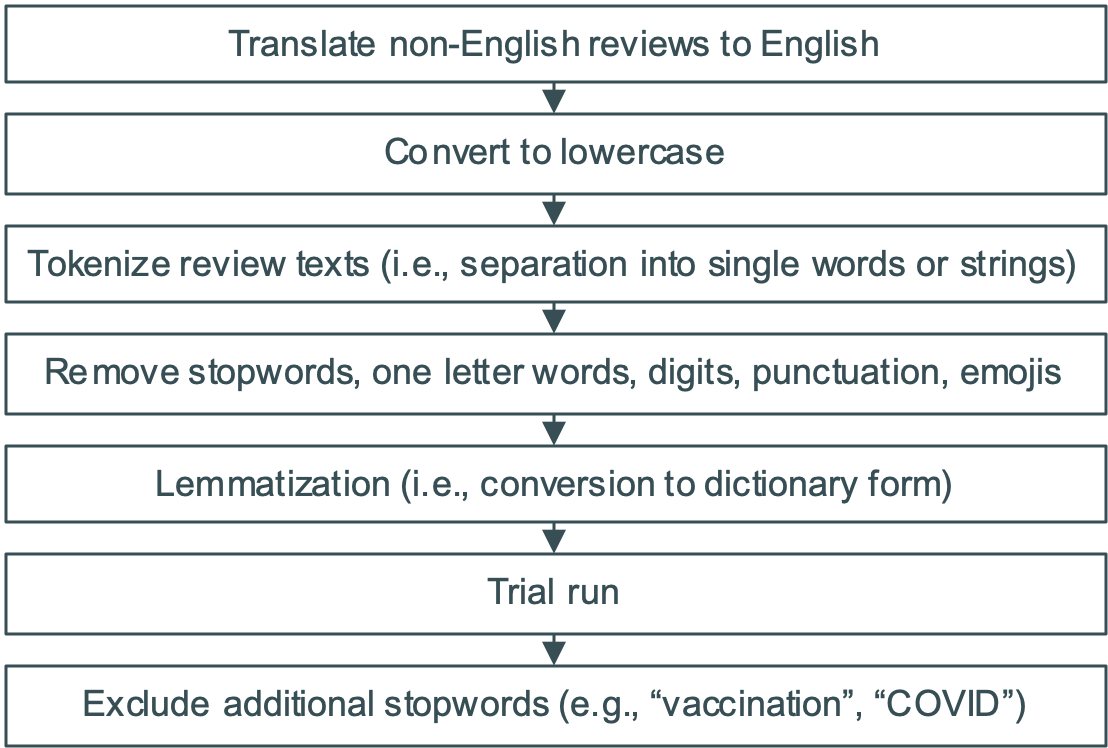


**Figure S1**: Data pre-processing procedure for LDA topic modeling

**Table S1:** Word grouping for keyword analysis

| **Keyword** | **Keywords to be grouped** |
| --- | --- |
| accompany | accompanied, accompany, accompanying |
| appointment | appointment, appointments |
| arrival | arrival, arrive, arrived, arriving |
| atmosphere | ambience, atmosphere, mood |
| bus | bus, buses |
| care | care, cared, caring |
| competent | competent, experienced |
| crowd | crowd, crowded, crowds, overcrowded |
| doctor | doctor, doctors, pediatricians, physician, vaccinator, vaccinators |
| entrance | entrance, entrances, entry |
| excited | excited, thrilled |
| experience | experience, experiences |
| feel | feel, feeling, feels, felt |
| friendliness | friendlier, friendliness, friendly |
| helpful | helpful |
| hour | hour, hours |
| long | long |
| military | army, bundeswehr, military, soldier, soldiers |
| minute | minutes |
| nice | nice |
| organization | organisation, organised, organization, organizational, organizationally, organizations, organize, organized, organizing |
| parking | carpark, parking |
| people | people |
| perfect | perfect, perfectly |
| place | place |
| pleasant | pleasant, pleasantly |
| polite | polite |
| process | procedure, procedures |
| professional | professional, professionalism, professionally, professionals, unprofessional |
| queue | line, lines, queue, queues, queued, queueing, snake |
| quick | fast, quick, quickly |
| recommend | recommend, recommendable, recommended |
| security | guard, guards, securitas, security, securitys |
| service | service, services |
| shuttle | shuttle, shuttles |
| smooth | smooth, smoothly |
| staff | employee, employees |
| station | station, stations |
| support | support |
| taxi | taxi, taxis |
| team | team, teams |
| thanks | appreciation |
| time | time, times |
| user | guests, patient, patients, user, users, vaccinee, vaccinees, visitor, visitors |
| wait | wait, waited, waiting |
| wheelchair | wheelchair, wheelchairs |
| work | work, works |

**Table S2:** Top 30 keywords from topic modeling by frequency and relevance.

| Topic | 1 | | 2 | | 3 | | 4 | | 5 | |
| --- | --- | --- | --- | --- | --- | --- | --- | --- | --- | --- |
| Label | Patient flow / scheduling / wait time | | Duration, efficiency, side effects | | Accessibility, experience | | Friendliness / responsiveness | | Site access, arrival, documentation | |
| Themes | Process | | Process, Vaccination | | Location | | Staff | | Process, location | |
| Top Terms | λ=1 | λ=0.6 | λ=1 | λ=0.6 | λ=1 | λ=0.6 | λ=1 | λ=0.6 | λ=1 | λ=0.6 |
| 1 | appointment | appointment | quick | quick | parking | event | friendly | friendly | minute | bus |
| 2 | hour | hour | start | start | location | location | organized | organized | parking | area |
| 3 | time | queue | side | finish | event | parking | staff | staff | entrance | entrance |
| 4 | minute | time | finish | healthy | entrance | uncomplicated | nice | nice | time | shuttle |
| 5 | queue | minute | healthy | side | see | welcome | super | super | waiting | parking |
| 6 | people | line | mask | mask | front | used | organization | thanks | area | certificate |
| 7 | waiting | wait | stay | effect | space | tip | thanks | organization | bus | car |
| 8 | line | day | effect | wish | uncomplicated | see | helpful | helpful | friendly | document |
| 9 | long | waiting | perfect | gate | like | concert | employee | thank | certificate | code |
| 10 | wait | long | looked | paper | used | effort | thank | employee | shuttle | digital |
| 11 | despite | outside | wish | gladly | building | parking | doctor | helper | people | qr |
| 12 | outside | people | gate | fact | experience | organized | people | doctor | car | minute |
| 13 | day | despite | processing | stay | hall | exception | time | quickly | digital | space |
| 14 | staff | booster | gladly | forever | welcome | mega | helper | people | document | exit |
| 15 | doctor | inside | support | competence | help | bike | quickly | time | code | german |
| 16 | booster | cold | paper | origin | worked | hall | waiting | process | space | simply |
| 17 | inside | moderna | smoothly | hurt | tip | street | process | felt | way | waiting |
| 18 | cold | least | medical | dad | effort | entrance | like | competent | appointment | english |
| 19 | nice | total | fact | word | organized | building | competent | fast | qr | different |
| 20 | moderna | waited | guy | ffp | free | organizer | work | team | exit | speak |
| 21 | back | stand | health | willingness | organization | spot | felt | extremely | like | way |
| 22 | organization | week | word | guy | employee | choose | fast | work | doctor | organize |
| 23 | least | biontech | hurt | comment | huge | incomprehensible | team | big | german | time |
| 24 | way | longer | easily | health | street | berliner | extremely | top | helper | something |
| 25 | site | completely | quickly | mutti | concert | deadline | big | like | station | according |
| 26 | organized | said | forever | easily | park | beautiful | top | perfectly | stimply | form |
| 27 | completely | end | competence | risk | young | space | perfectly | waiting | something | station |
| 28 | employee | standing | willingness | arrogant | people | front | service | incredibly | process | including |
| 29 | total | doctor | risk | processing | exception | ever | perfect | perfect | including | ask |
| 30 | longer | back | origin | velodrome | steward | parked | smoothly | feel | quickly | small |

**Figure S2**: Keywords contained in at least 1% of reviews sorted by key determinant.
